# Supplementary material for: Carboxymethyl Cellulose Entrapped in a Poly(vinyl) Alcohol Network: Plant-Based Scaffolds for Cartilage Tissue Engineering
Source: Molecules. 2021 Jan 22;26(3):578. doi: 10.3390/molecules26030578 (PMC7865723; doi:10.3390/molecules26030578)
Supplement: Supplementary file 1 [file molecules-26-00578-s001.pdf]

Supplementary Materials

Carboxymethyl Cellulose Entrapped in a Poly(vinyl) Alcohol Network: Plant-based Scaffolds for Cartilage Tissue Engineering

Jirapat Namkaew[1], Panitporn Laowpanitchakorn[2] , Nuttapong Sawaddee[1] , Sirinee Jirajessada[3], Sittisak Honsawek[4] and Supansa Yodmuang[5,*]

[1] Excellence center for Advanced therapy Medicinal Products, King Chulalongkorn Memorial Hospital, Pathumwan, Bangkok 10330, Thailand; namkaew.j@gmail.com (J.N.); nuttapong_tontoey@hotmail.com (N.S.)
[2] Biomaterial Engineering for Medical and Health Research Unit, Chulalongkorn University, Pathumwan, Bangkok 10330, Thailand; antpnp@gmail.com (P.L.)
[3] Biology program, Faculty of Science, Buriram Rajabhat University, Muang, Buriram 31000, Thailand; sirinee.ym@bru.ac.th (S.J.)
[4] Osteoarthritis and Musculoskeleton Research Unit, Faculty of Medicine, Chulalongkorn University, Pathumwan, Bangkok 10330, Thailand; sittisak.h@chula.ac.th (S.H.)
[5] Research Affairs, Faculty of Medicine, Chulalongkorn University, Pathumwan, Bangkok 10330, Thailand; supansa.y@chula.ac.th (S.Y.)

* Correspondence: Supansa Yodmuang
Research Affairs, Anunda Mahidol Building
Faculty of Medicine, Chulalongkorn University
1873 Rama 4 Rd, Pathumwan, Bangkok 10330 Thailand
supansa.y@chula.ac.th; Tel: +66-6-3774-8604

Figure S1: Glass transition (T_g), melting temperature (T_m), and crystallinity (T_c) of PVA/CMC scaffolds analyzed by DSC technique. P1C0 is PVA/CMC = 1:0; P5C1 is PVA/CMC = 5:1; P3C1 is PVA/CMC = 3:1

Groups	GA/PVA crosslink ratio	2nd heat Tg (°C)	2nd heat Tm (°C)	cooling down Tc (°C)
**P1C0**	1:0.4	84.2	-	97.4
	1:0.2	78.1	137.3	126.9
	1:0.1	72.0	156.5	142.4
**P5C1**	1:0.4	84.6	-	-
	1:0.2	75.8	144.1	136.3
	1:0.1	70.5	154.6	133.2
**P3C1**	1:0.4	81.5	-	136.6
	1:0.2	76.0	145.9	135.9
	1:0.1	69.1	157.7	136.6

Figure S2: Young's modulus (kPa) of PVA/CMC scaffold without cell seeding. Data show average ± standard error; n=4

Group	Crosslinking ratio		
	**GA/PVA = 0.4**	**GA/PVA = 0.2**	**GA/PVA = 0.1**
**P1C0 (PVA control)**	344.735 ± 52	322.646 ± 26.18	175.4 ± 24.31
**P5C1**	287.605 ± 38.57	101.86 ± 12.21	23.73 ± 5.93
**P3C1**	108.98 ± 7.63	81.35 ± 14	14.77 ± 1.49

Figure S3: In vitro scaffold degradation of PVA/CMC scaffolds in PBS solution
(A) P1C0 control scaffolds (PVA/CM = 1:0); **(B)** P5C1 scaffolds (PVA/CMC = 5:1); **(C)** P3C1 scaffolds (PVA/CMC = 3:1). Data show average ± standard error, n=8.

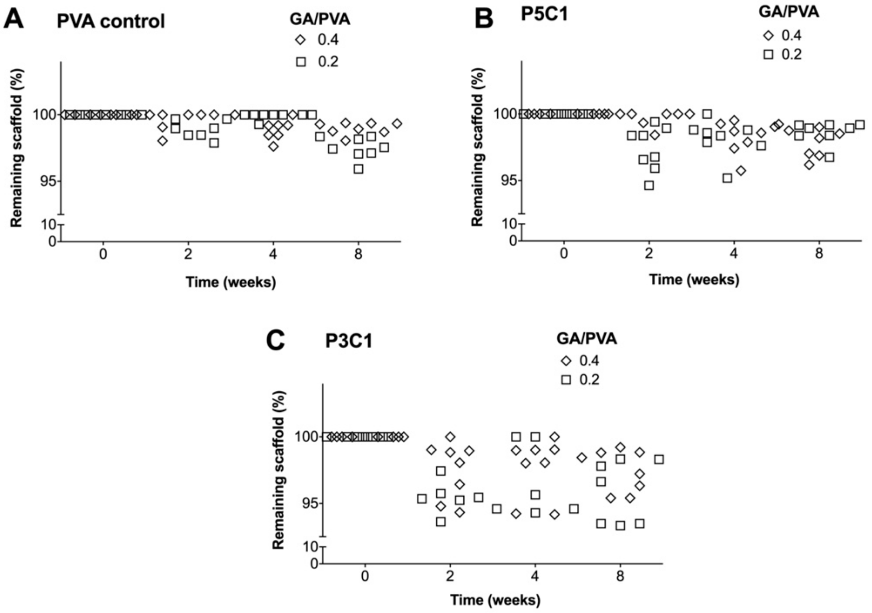

Scaffolds were soaked in sterile PBS solution at 37°C to determine remaining weight for 8 weeks, the time course used in vitro cartilage tissue development. The initial weight of dry scaffolds (W_i) was recorded. At the end of each time point, the scaffolds were removed from PBS solution, washed with distilled water 3 times, 5 minutes each, and dried in 80°C hot air oven. The weight of final dry scaffolds (W_f) was measured to calculate percentage of scaffold remaining weight in equation

$$\text{Remaining Weight} = W_f / W_i \times 100$$

All groups, scaffolds at GA:PVA crosslinking ratio of 0.4 : 1 and 0.2 : 1 were chosen to determined remaining weights. At the end of 8 weeks, slight weight loss was observed in all groups.

	**% Remaining weight**	
**Groups**	**GA:PVA = 0.4:1**	**GA:PVA = 0.2 : 1**
**P1C0 (PVA control)**	98.9%	97.5%
**P5C1**	97.9%	98.2%
**P3C1**	97.3%	95.3%

Figure S4: Maximum swelling ratios of P1C0 (PVA control), P5C1 and P3C1 scaffolds that did not proceed to the freeze-drying step. Scaffolds were dried at 60 °C in a hot air oven for 48 hours and soaked in PBS solution for 24 hours

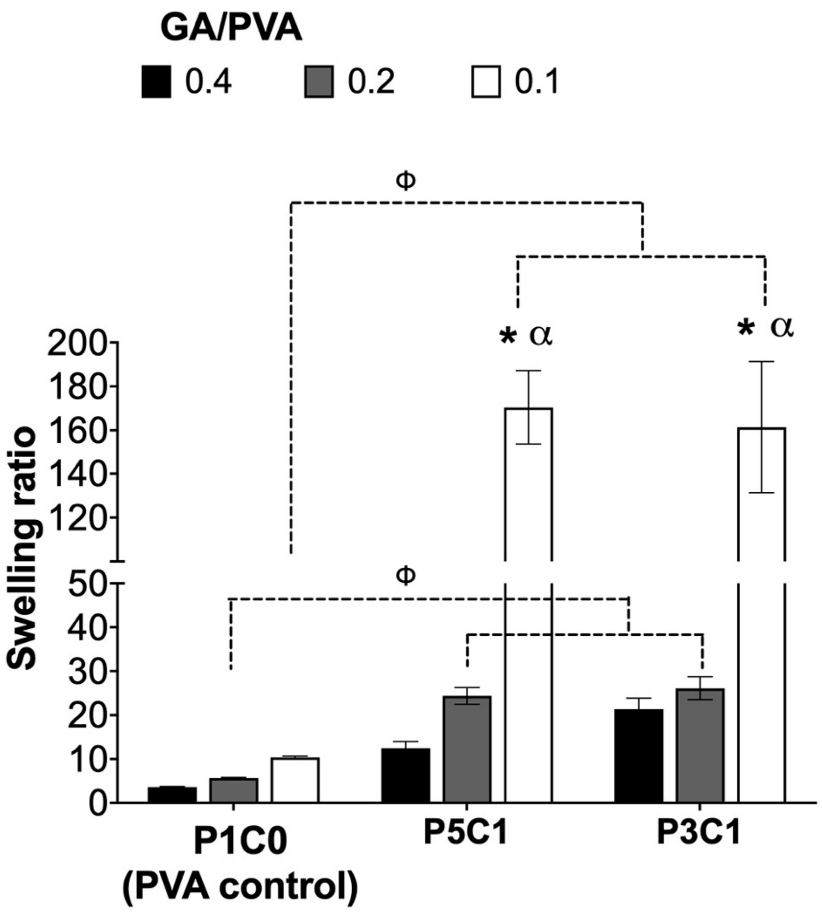

Data show average ± standard error; n = 4. $^{\phi}$ indicates significant effects of CMC. * and $^{\alpha}$ indicate significant effects of crosslinking compared with GA/PVA crosslinking ratios of 0.4 and 0.2, respectively. P1C0 is PVA/CMC = 1:0; P5C1 is PVA/CMC = 5:1; P3C1 is PVA/CMC = 3:1.)

Figure S5: Water content of scaffolds

Water content = $(W_w - W_d)/W_w \times 100$
W_w = wet weight
W_d = dry weight

PVA/CMC = 1:0 **P1C0 (PVA control)**

**GA/PVA = 0.4**	Weight (g)			
**Scaffolds (n=4)**	**1**	**2**	**3**	**4**
Dry weight, W_d (g)	13.60	13.25	12.85	13.60
Wet weight, W_w (g)	42.10	42.00	38.80	41.40
water (g)	28.50	28.75	25.95	27.80
water content (%)	67.70	68.45	66.88	67.15
**Average water content (%)**	67.54			

**GA/PVA = 0.2**	Weight			
**Scaffolds (n=4)**	**1**	**2**	**3**	**4**
Dry weight, W_d (g)	13.00	10.80	11.70	11.23
Wet weight, W_w (g)	51.20	44.50	41.90	42.60
water (g)	38.20	33.70	30.20	31.37
water content (%)	74.61	75.73	72.08	73.64
**Average water content (%)**	74.01			

**GA/PVA = 0.1**	Weight			
**Scaffolds (n=4)**	**1**	**2**	**3**	**4**
Dry weight, W_d (g)	10.50	9.65	10.05	9.75
Wet weight, W_w (g)	43.60	42.90	42.60	41.80
water (g)	33.10	33.25	32.55	32.05
water content (%)	75.92	77.51	76.41	76.67
**Average water content (%)**	76.63			

**GA/PVA = 0.05**	Weight			
**Scaffolds (n=4)**	**1**	**2**	**3**	**4**
Dry weight, W_d (g)	6.10	6.30	7.10	6.40
Wet weight, W_w (g)	111.60	88.00	98.40	119.60
water (g)	105.50	81.70	91.30	113.20
water content (%)	94.53	92.84	92.78	94.65
**Average water content (%)**	93.70			

PVA/CMC = 5:1

GA/PVA = 0.4	Weight			
**Scaffolds (n=4)**	**1**	**2**	**3**	**4**
Dry weight, W_d (g)	7.46	8.62	8.60	7.15
Wet weight, W_w (g)	24.00	25.90	25.50	25.40
water (g)	16.54	17.28	16.90	18.25
water content (%)	68.92	66.72	66.27	71.85
**Average water content (%)**	68.44			

GA/PVA = 0.2	Weight			
**Scaffolds (n=4)**	**1**	**2**	**3**	**4**
Dry weight, W_d (g)	6.38	7.55	7.43	8.63
Wet weight, W_w (g)	24.30	24.30	28.00	24.60
water (g)	17.93	16.75	20.57	15.97
water content (%)	73.77	68.93	73.46	64.92
**Average water content (%)**	70.27			

GA/PVA = 0.1	Weight			
**Scaffolds (n=4)**	**1**	**2**	**3**	**4**
Dry weight, W_d (g)	4.00	4.00	1.90	2.40
Wet weight, W_w (g)	52.30	49.40	25.60	38.70
water (g)	48.30	45.40	23.70	36.30
water content (%)	92.35	91.90	92.58	93.80
**Average water content (%)**	92.66			

PVA/CMC = 3:1

GA/PVA = 0.4	Weight			
**Scaffolds (n=4)**	**1**	**2**	**3**	**4**
Dry weight, W_d (g)	6.40	5.60	5.40	4.40
Wet weight, W_w (g)	19.90	20.40	19.40	18.30
water (g)	13.50	14.80	14.00	13.90
water content (%)	67.84	72.55	72.16	75.96
**Average water content (%)**	72.13			

GA/PVA = 0.2	Weight			
**Scaffolds (n=4)**	**1**	**2**	**3**	**4**
Dry weight, W_d (g)	4.30	5.90	6.35	6.45
Wet weight, W_w (g)	23.80	24.30	33.80	32.70
water (g)	19.50	18.40	27.45	26.25

water content (%)	81.93	75.72	81.21	80.28

Average water content (%) 79.79

**GA/PVA = 0.1**	Weight			
**Scaffolds (n=4)**	**1**	**2**	**3**	**4**
Dry weight, W_d (g)	2.60	3.36	2.95	2.75
Wet weight, W_w (g)	35.20	40.70	32.00	32.80
water (g)	32.60	37.34	29.05	30.05
water content (%)	92.61	91.74	90.78	91.62

Average water content (%) 91.69

Figure S6: Solid-state NMR spectra of P1C0 control group at GA/PVA crosslink ratio of 0.4 and P3C1 scaffold at GA/PVA crosslinking ratio of 0.4. Carbon atoms in CMC are indicated by number 1 to 8. Carbon atoms in PVA are indicated by a to e

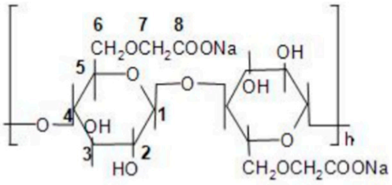

CMC

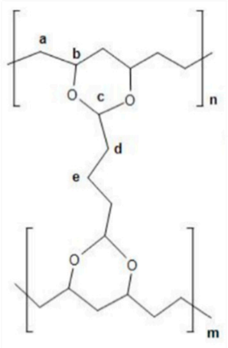

PVA crosslinked GA

The ^{13}C CP/MAS NMR spectra of PVA and PVA-CMC crosslinked with GA

The P1C0 PVA control group: From previous research, pure PVA has the ^{13}C-NMR chemical shift in the range of 30-40 ppm corresponding to methylene C_d, and the range of 60-40 ppm corresponding to C_b, which attaches to hydroxyl group [1]. Since polyvinyl alcohol is produced commercially from polyvinyl acetate, the peak at 171.69 ppm accounts for the carbonyl groups (-C=O-) of vinyl acetate remained in polyvinyl alcohol [2]. The formation of new acetal bridge (C_c) was recorded at 101.64 ppm [3]. The peak at 21.44 ppm is attributed to the methylene C_e from glutaraldehyde [4].

P1C0 at GA/PVA crosslinking ratio of 0.4 (PVA control)

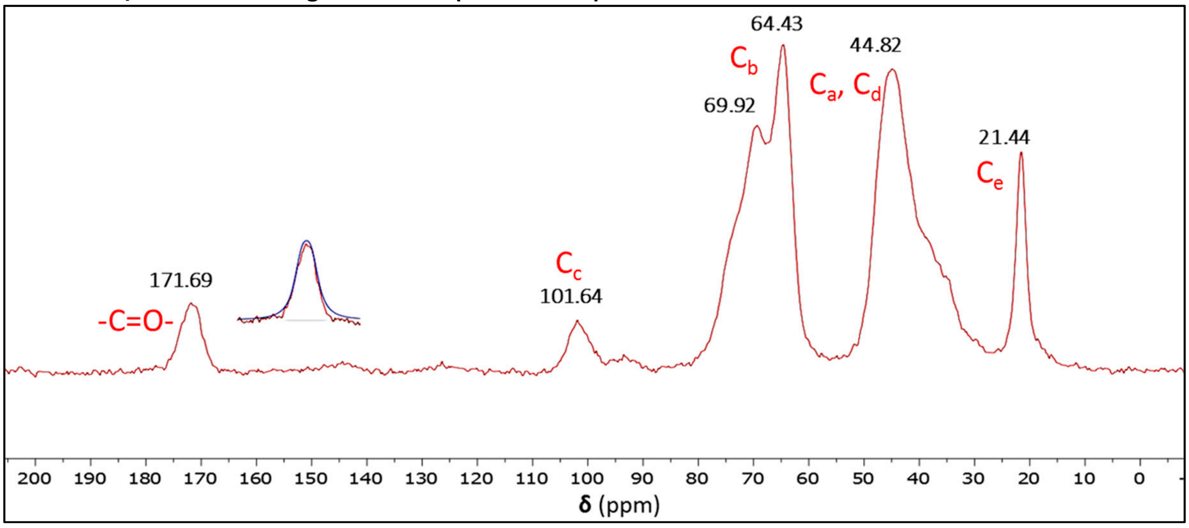

The PVA/CMC scaffolds, P3C1 with GA/PVA crosslinking ratio of 0.4: The spectrum of CMC is assigned in the region of 180-170, 105-100, and 90-60 ppm, corresponding to carbonyl groups (-COO-), C_1 and the carbons in the 2-7 positions [5]. The spectrum collected from ^{13}C CP/MAS NMR is broader than liquid-state ^{13}C-NMR. Therefore, the results show an overlapped region of PVA and CMC. There is no difference observed between P1C0 and P3C1 scaffolds in the region of 20-105 ppm. The significant carbonyl group of CMC (C_8) was detected with carbonyl groups of vinyl acetate in PVA at 170.37 and 172.37 ppm, respectively. Comparing P1C0 and P3C1 spectrum, there is only the resonance of acetal bridge at 101 ppm indicating the crosslinking between PVA and GA. There is no signal confirming the reaction between CMC and GA.

P3C1 at GA/PVA crosslinking ratio of 0.4

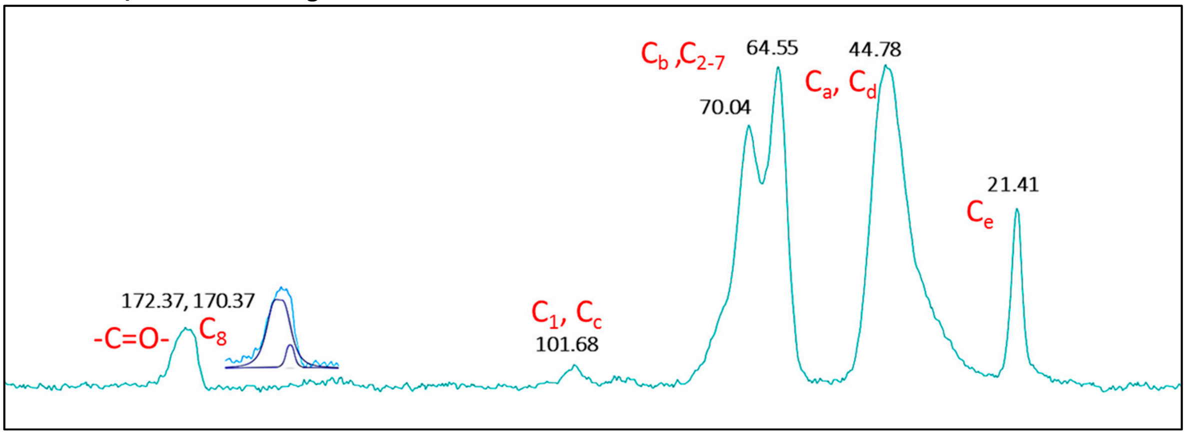

References

1. Lai, S., et al., *Solid-State 13C NMR Study of Poly(vinyl alcohol) Gels.* Solid state nuclear magnetic resonance, 2002. 21: p. 187-96.
2. Mjakin, S.V., et al., *Improvement of vibrodamping properties of polyvinyl acetate-graphite composites by electron beam processing of the filler.* Springerplus, 2016. 5(1): p. 1539.
3. Doll, K.M. and S.Z. Erhan, *Synthesis of cyclic acetals (ketals) from oleochemicals using a solvent free method.* Green Chemistry, 2008. 10(6): p. 712-717.
4. Kildeeva, N., et al., *About mechanism of chitosan cross-linking with glutaraldehyde.* 2009. 35: p. 360-369.
5. Kono, H.J.C.p., *Characterization and properties of carboxymethyl cellulose hydrogels crosslinked by polyethylene glycol.* 2014. 106: p. 84-93.

P1C0 at GA/PVA crosslinking ratio of 0.4 (PVA control)

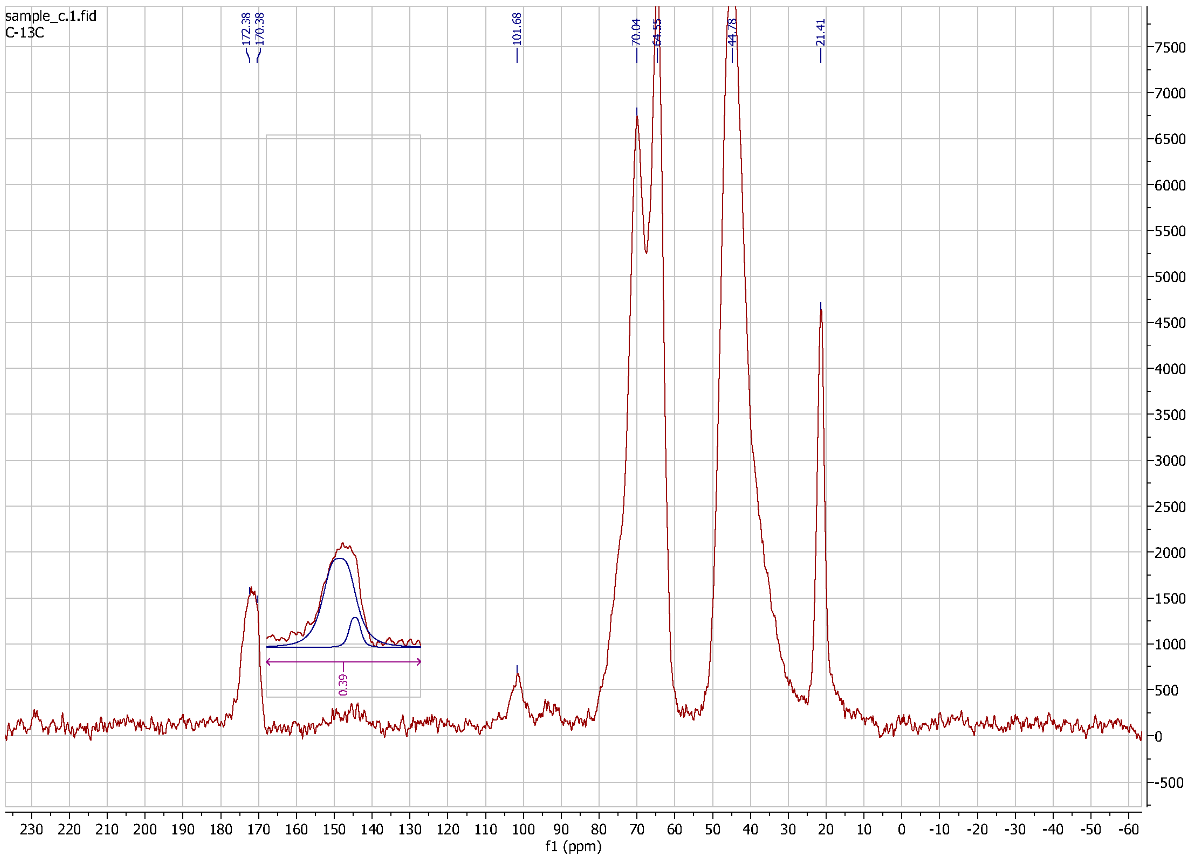

P3C1 at GA/PVA crosslinking ratio of 0.4

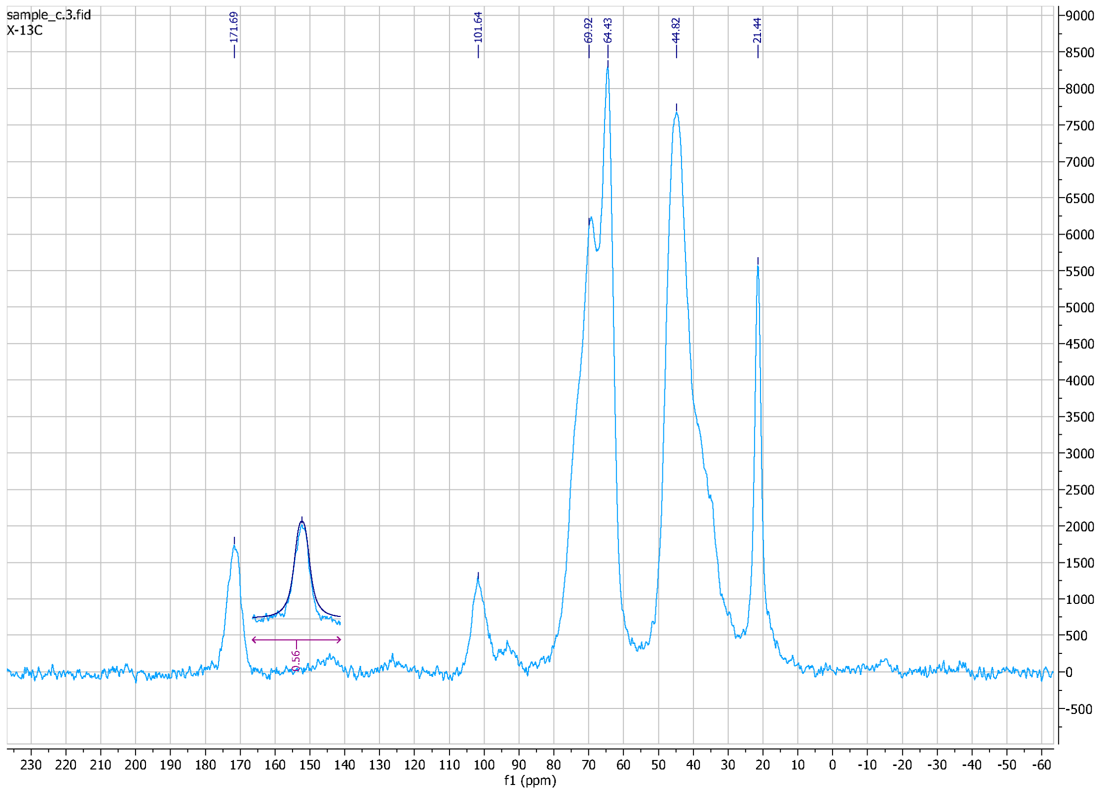

Figure S7: Preparation of porous of PVA/CMC scaffolds

PVA MW = 44.05

Glutaraldehyde MW = 100.11

P1C0

(PVA control group)

PVA/ CMC = 1:0 by mass

PVA = 6.25 g mole of 6.25 g PVA = 0.14

CMC = 0 g

12.5 %(w/v) PVA total volume of PVA hydrogel = 50 ml

mole PVA	mole GA	GA/PVA mole ratio	gram of GA	volume of GA from 25% (ml)	Volume of water (ml)	total volume (ml)
0.14	0.0560	0.4	5.60616	22.42464	27.57536	50
0.14	0.0280	0.2	2.80308	11.21232	38.78768	50
0.14	0.0140	0.1	1.40154	5.60616	44.39384	50
0.14	0.0035	0.05	0.350385	1.40154	48.59846	50

P5C1

PVA/ CMC = 5:1 by mass

PVA = 6.25 g mole of 6.25 g PVA = 0.14

CMC = 1.25 g

12.5 %(w/v) PVA total volume of hydrogel mixture = 50 ml

2.5 %(w/v) CMC

mole PVA	mole GA	GA/PVA mole ratio	gram of GA	volume of GA from 25% (ml)	Volume of water (ml)	total volume (ml)
0.14	0.0560	0.4	5.60616	22.42464	27.57536	50
0.14	0.0280	0.2	2.80308	11.21232	38.78768	50
0.14	0.0140	0.1	1.40154	5.60616	44.39384	50
0.14	0.0035	0.05	0.350385	1.40154	48.59846	50

P3C1

PVA/ CMC = 3:1 by mass

PVA = 6.25 g mole of 6.25 g PVA = 0.14

CMC = 2.09 g

12.5 %(w/v) PVA total volume of hydrogel mixture = 50 ml

4.17 %(w/v) CMC

mole PVA	mole GA	GA/PVA mole ratio	gram of GA	volume of GA from 25% (ml)	Volume of water (ml)	total volume (ml)
0.14	0.0560	0.4	5.60616	22.42464	27.57536	50
0.14	0.0280	0.2	2.80308	11.21232	38.78768	50
0.14	0.0140	0.1	1.40154	5.60616	44.39384	50
0.14	0.0035	0.05	0.350385	1.40154	48.59846	50
